# *Ixodes scapularis* Tick Parasitizing Dog in Dawson County, Montana, USA, 2023

**DOI:** 10.3201/eid3102.241308

**Published:** 2025-02

**Authors:** Philip E. Stewart, Justin B. Lack, Marni Rolston, Kimmo Virtaneva, Paul A. Beare, Craig M. Martens, Marshall E. Bloom, Tom G. Schwan

**Affiliations:** National Institutes of Health Rocky Mountain Laboratories, Hamilton, Montana, USA (P.E. Stewart, K. Virtaneva, P.A. Beare, C.M. Martens, M.E. Bloom, T.G. Schwan); National Institutes of Health, Rockville, Maryland, USA (J.B. Lack); Montana State University, Bozeman, Montana, USA (M. Rolston)

**Keywords:** *Ixodes scapularis*, blacklegged tick, Ixodidae, Lyme disease, dog, cytochrome oxidase C subunit 1, *cox1*, *Rickettsia buchneri*, bacteria, *Rickettsia*, vector-borne infections, zoonoses, Montana, United States, ticks

## Abstract

In October 2023, a partially engorged female *Ixodes* tick was removed from a dog in Bozeman, Montana, USA, that had recently spent time in eastern Montana. The tick was identified as *I. scapularis* according to morphologic characteristics and genomic sequencing, suggesting an expanded geographic distribution requiring continued public health surveillance.

The blacklegged tick, *Ixodes scapularis*, is the vector of several human pathogens and the primary vector of the Lyme disease spirochete *Borrelia burgdorferi* in the eastern half of the continental United States (*1*). During the past 40 years, intensive investigations of this tick’s geographic distribution have documented its spread north and west, with new populations established in eastern North and South Dakota (*2**,**3*). Montana has remained free of *I. scapularis* ticks and their western counterparts, *I. pacificus* ticks (*2*). As part of a citizen science investigation during 2016–2017, a specimen of *I. scapularis* tick was submitted from Liberty County, Montana; however, the tick stage and host were not given, and the authors concluded that either the tick had been recently imported to the area or had been misidentified (*4*).

On October 12, 2023, a partially engorged female *Ixodes* tick was removed from the lower neck of a 7.5-year-old female French Brittany hunting dog in Bozeman, Montana, USA; the dog had recently returned with its owners from a pheasant hunting trip north of Ritchey, Dawson County, in eastern Montana. The specimen was submitted to the Schutter Diagnostic Laboratory at Montana State University in Bozeman for identification, and subsequently stored in alcohol and forwarded to the Rocky Mountain Laboratories, National Institutes of Health (Hamilton, MT, USA) for further examination. The specimen lacked the hypostome and 1 palp but, when examined microscopically, the characteristics were consistent with an *I. scapularis* tick (Figure 1, panels A–D). The specimen and 3 other museum specimens of unengorged female *I. scapularis* ticks matched *I. scapularis* (*5**,**6*) but not any of the 9 *Ixodes* spp. previously recorded in Montana (Appendix Table). The dog had not traveled outside the state before acquiring the tick, and the presumed time of attachment correlated with the trip to Dawson County (Appendix Figure 1).

**Figure 1:**
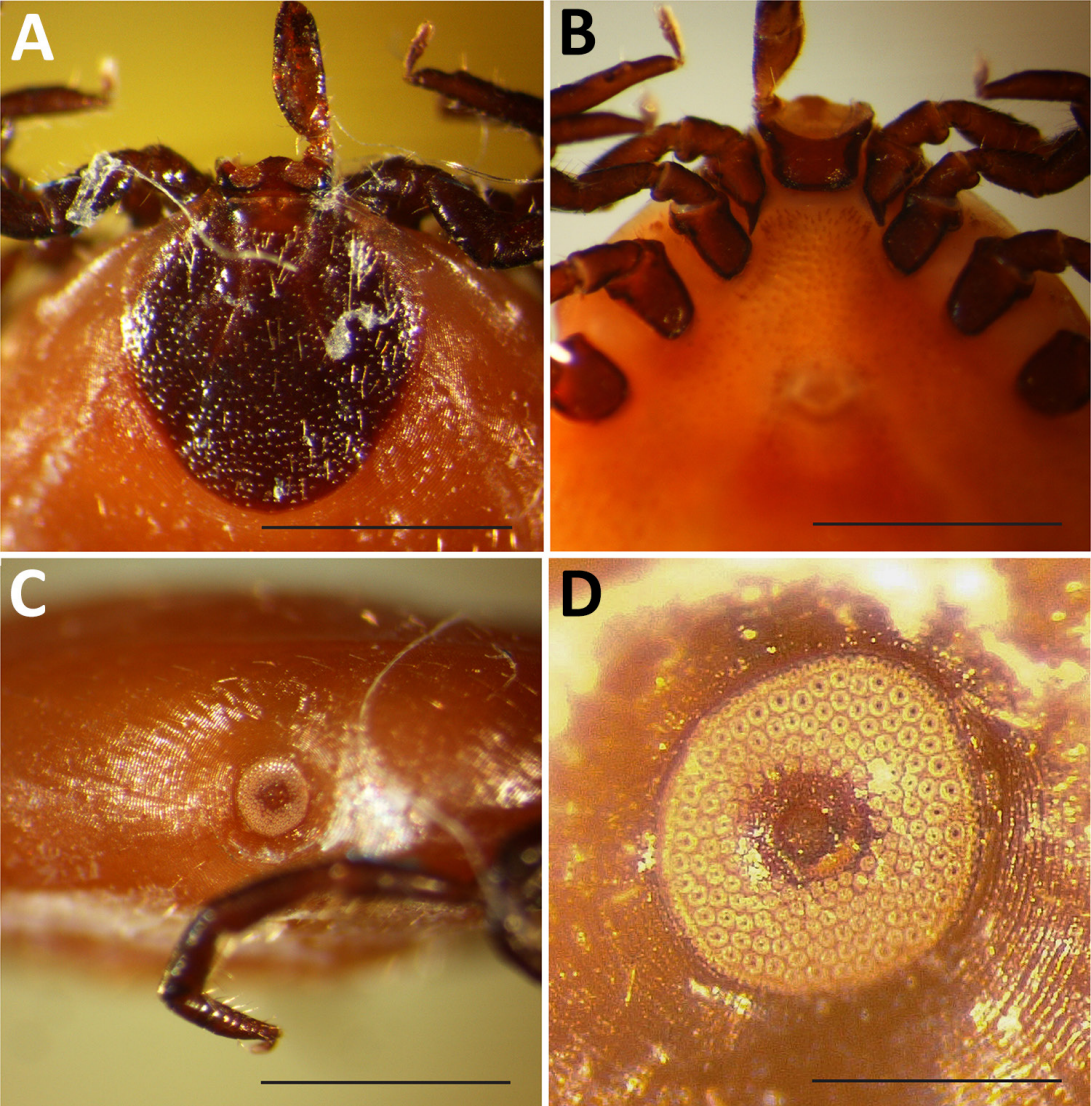
Partially engorged female *Ixodes scapularis* tick from Dawson County, Montana, USA, 2023. Tick was found on a dog, and key characteristics were used for identification. A) Dorsal view of capitulum showing shape of scutum and palp, basis capituli, and porose areas. Scale bar = 1 mm. B) Ventral view of capitulum showing shape and length of internal spurs on coxae I. Scale bar = 1 mm. C) Right lateral view of idiosoma showing spiracular plate. Scale bar = 1 mm. D) Enlargement of spiracular plate showing number and size of the goblet cells. Scale bar = 0.25 mm.

Because of the condition of the specimen and potential significance of its identity, we processed the tick for genomic sequencing. We removed the tick from alcohol, froze it in liquid nitrogen, then pulverized it and extracted genomic DNA by using the MagAttract HMW DNA Kit (QIAGEN, https://www.qiagen.com) according to the manufacturer’s protocol. DNA isolated from this tick was partially degraded and produced a low yield, likely because of the long-term storage in alcohol; however, we performed whole-genome sequencing.

Sequencing coverage was ≈1× for the nuclear genome; the median mitochondrial genome coverage mapped to the published *I. scapularis* mitochondrial DNA sequence (GenBank accession no. MZ645749.1) (*7*). We extracted the *cox1* gene consensus sequence from the data and generated a maximum-likelihood phylogeny that had 100% bootstrap support for identity between *I. scapularis* and this specimen. The uncorrected pairwise identity between *I. scapularis* and this specimen was 99.49%, whereas pairwise identity with several other *Ixodes* spp. established in Montana was <89% (Figure 2). We deposited the *cox1* sequence into GenBank (accession no. PQ284574).

**Figure 2:**
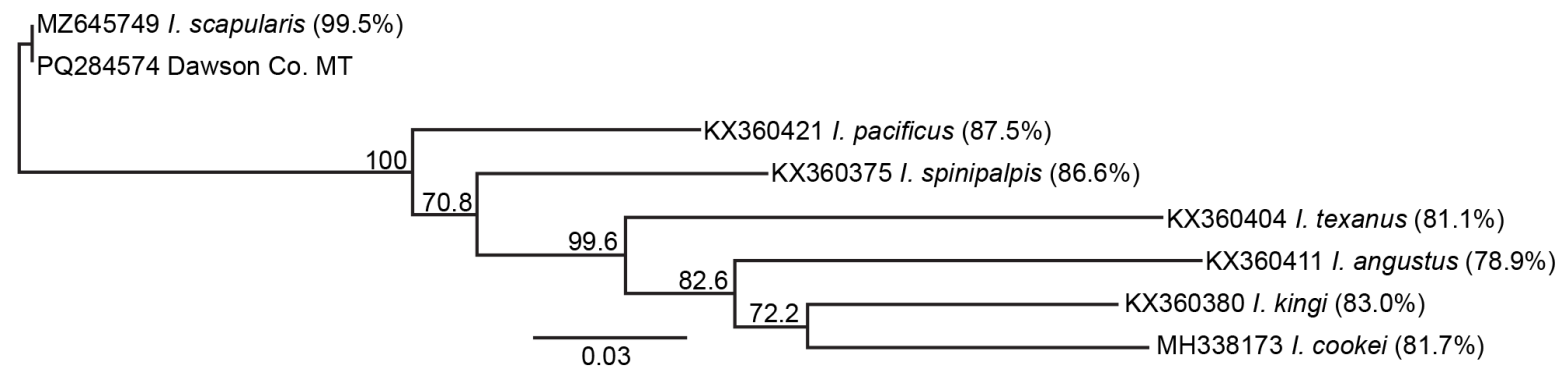
Phylogenetic analysis of *Ixodes scapularis* tick parasitizing dog in Dawson County, Montana, USA, 2023. Phylogram of cytochrome oxidase C subunit I (*coxI*) sequences showing near identity of the Dawson County tick with *I. scapularis*. We used RAxML (https://github.com/amkozlov/raxml-ng) to generate an unrooted maximum-likelihood phylogeny of 583-bp *coxI* sequences with 500 bootstrap replicates. Genbank accession numbers for each sequence precede the species name. Branch labels indicate the percentage of bootstrap replicates supporting a given branch. Percentages of uncorrected pairwise identity to the Dawson County, Montana, specimen are indicated in parentheses. With the exceptions of *I. cookei* and *I. pacificus*, the other *Ixodes* spp. ticks in this tree are indigenous to Montana. Scale bar indicates nucleotide substitutions per site.

As an independent approach separate from mapping to the *I. scapularis* reference genome, we mapped the Illumina sequencing reads from this specimen to a database of >5 million *cox1* sequences (*8*). The highest count (6,174,692 reads) mapped to *I. scapularis*, far more than the next highest count, which was *I. ricinus* (718,590 reads). In addition, we analyzed the microbial sequences from this tick specimen and identified 340,000 reads mapping to the *I. scapularis*–specific endosymbiont bacterium *Rickettsia buchneri* (Appendix Figure 2) (*9*), further supporting the identification of this tick. A relatively low number (1,509) of reads mapped to the family Borreliaceae, which includes the causative agents of Lyme disease and relapsing fever. Although this low number of reads requires independent verification, it suggests the tick specimen might have been infected with a member of the genus *Borrelia*.

In conclusion, the continued range expansion of *I. scapularis* ticks is thought to be complex and multifactorial (*10*). Here, we confirmed that a tick acquired in eastern Montana was *I. scapularis* by using 2 independent methods: morphologic characterization and genomic DNA sequence alignments. Identification is further supported by the presence of *R. buchneri* in the tick’s microbiome. In 2023, the Centers for Disease Control and Prevention predicted the western boundary of the *I. scapularis* tick range terminated in the middle of North Dakota (Appendix Figure 1). The collection of a single specimen does not signify an established population in Montana, but the public health concern regarding this tick species warrants further investigation into the potential range expansion of the *I. scapularis* tick. If an established *I. scapularis* tick population is confirmed, continued surveillance and characterization of any corresponding human pathogens will be required. 

Appendix Additional information for *Ixodes scapularis* tick parasitizing dog in Dawson County, Montana, USA, 2023.
