# Supplementary material for: Ixodes scapularis Tick Parasitizing Dog in Dawson County, Montana, USA, 2023
Source: Emerg Infect Dis. 2025 Feb;31(2):404–6. doi: 10.3201/eid3102.241308 (PMC11845153; doi:10.3201/eid3102.241308)
Supplement: Appendix — Additional information for Ixodes scapularis tick parasitizing dog in Dawson County, Montana, USA, 2023. [file 24-1308-Techapp-s1.pdf]

Article DOI: https://doi.org/10.3201/eid3102.241308

EID cannot ensure accessibility for supplementary materials supplied by authors. Readers who have difficulty accessing supplementary content should contact the authors for assistance.

Ixodes scapularis Tick Parasitizing Dog in Dawson County, Montana, USA, 2023

Appendix

The *Ixodes* ticks in Montana are 3-host species, each life stage feeding on a different individual host. Most of those ticks parasitize small- to medium-sized rodents, small carnivores, lagomorphs including rabbits and pikas, and, in 1 case, birds (Appendix Table) (*1–4*). Humans rarely encounter those ticks unless they are hunting or trapping the ticks' hosts. Only a single nymph of 1 species (*I. spinipalpis*) has been recovered from a human in Montana (*5*), which likely resulted from the individual hunting rabbits or hares. Similar to *I. scapularis* in this report, 1 other species, *I. kingi*, has been recovered on rare occasions from domestic dogs.

References

1. Cooley RA, Kohls GM. The genus *Ixodes* in North America. Natl Inst Health Bull No. 184. Washington: US Government Printing Office; 1945

2. Keirans JE, Clifford CM. The genus *Ixodes* in the United States: a scanning electron microscope study and key to the adults. J Med Entomol. Suppl. 1978;2:1–149. PubMed https://doi.org/10.1093/jmedent/15.suppl2.1

3. Kohls GM. Notes on the tick, *Ixodes howelli* Cooley and Kohls, with descriptions. J Parasitol. 1947;33:57–61. PubMed https://doi.org/10.2307/3273621

4. Durden LA, Keirans JE. 1996. Nymphs of the genus *Ixodes* (Acari: Ixodidae) of the United States: taxonomy, identification key, distribution, hosts, and medical/veterinary importance. Lanham, Maryland: Entomological Society of America; 1996

5. Merten HA, Durden LA. A state-by-state survey of ticks recorded from humans in the United States. J Vector Ecol. 2000;25:102–13. PubMed

6. Wood DE, Lu J, Langmead B. Improved metagenomic analysis with Kraken 2. Genome Biol. 2019;20:257. PubMed https://doi.org/10.1186/s13059-019-1891-0

7. Kurtti TJ, Felsheim RF, Burkhardt NY, Oliver JD, Heu CC, Munderloh UG. *Rickettsia buchneri* sp. nov., a rickettsial endosymbiont of the blacklegged tick *Ixodes scapularis.* Int J Syst Evol Microbiol. 2015;65:965–70. PubMed https://doi.org/10.1099/ijs.0.000047

Appendix Table. Species of *Ixodes* ticks and their recorded hosts in Montana

Species	Hosts in Montana
Ixodes angustus	Red squirrel (*Tamiasciuris hudsonicus*), bushy-tailed woodrat (*Neotoma cinerea*)
I. hearlei	Golden mantled ground squirrel (*Callospermophilus lateralis*)
I. howelli	Cliff swallow (*Petrochelidon pyrrhonota*)
I. kingi	Carnivores, rodents, dogs
I. marmotae	Yellow-bellied marmot (*Marmota flaviventris*)
I. ochotonae	Pika (*Ochotona princeps*)
I. sculptus	Ground squirrels (*C. lateralis, Urocitellus* spp.), northern pocket gopher (*Thomomys talpoides*)
I. spinipalpus	Mountain cottontail rabbit (*Sylvilagus nuttallii*)
I. texanus	American marten (*Martes americana*), weasels (*Mustella* spp.), golden mantled ground squirrels (*C. lateralis*)

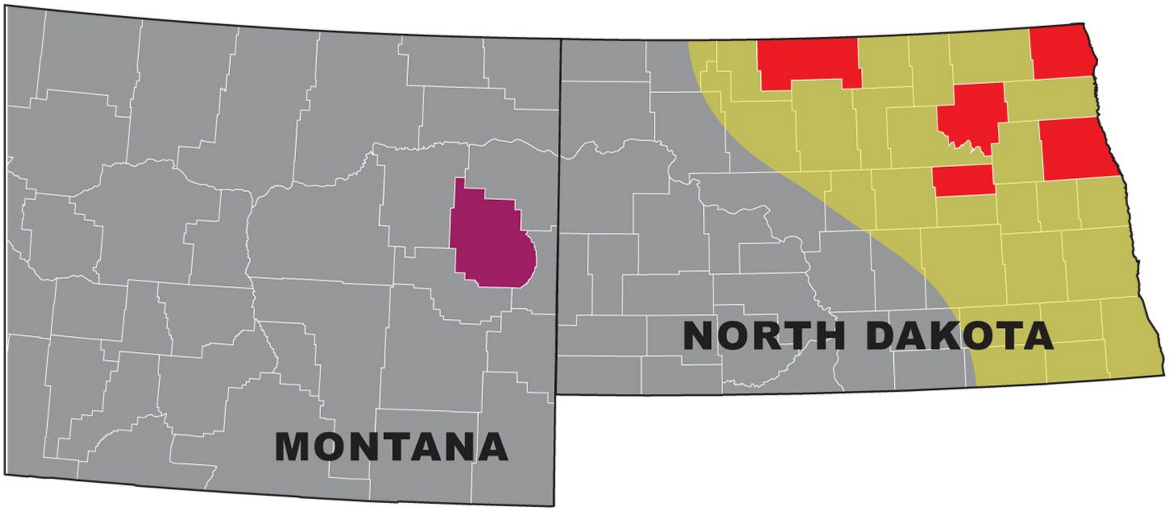

Appendix Figure 1. Location of *Ixodes scapularis* specimen in study of tick parasitizing dog in Dawson County, Montana, USA, 2023. Map of North Dakota and eastern Montana indicates Dawson County (purple), where the tick specimen originated and 5 counties in North Dakota that have established populations of *I. scapularis* (red). Yellow shading indicates the distribution of this tick species predicted by the Centers for Disease Control and Prevention in 2023, and the map was derived from their website showing *I. scapularis* surveillance (https://www.cdc.gov/ticks/data-research/facts-stats/blacklegged-tick-surveillance.html).

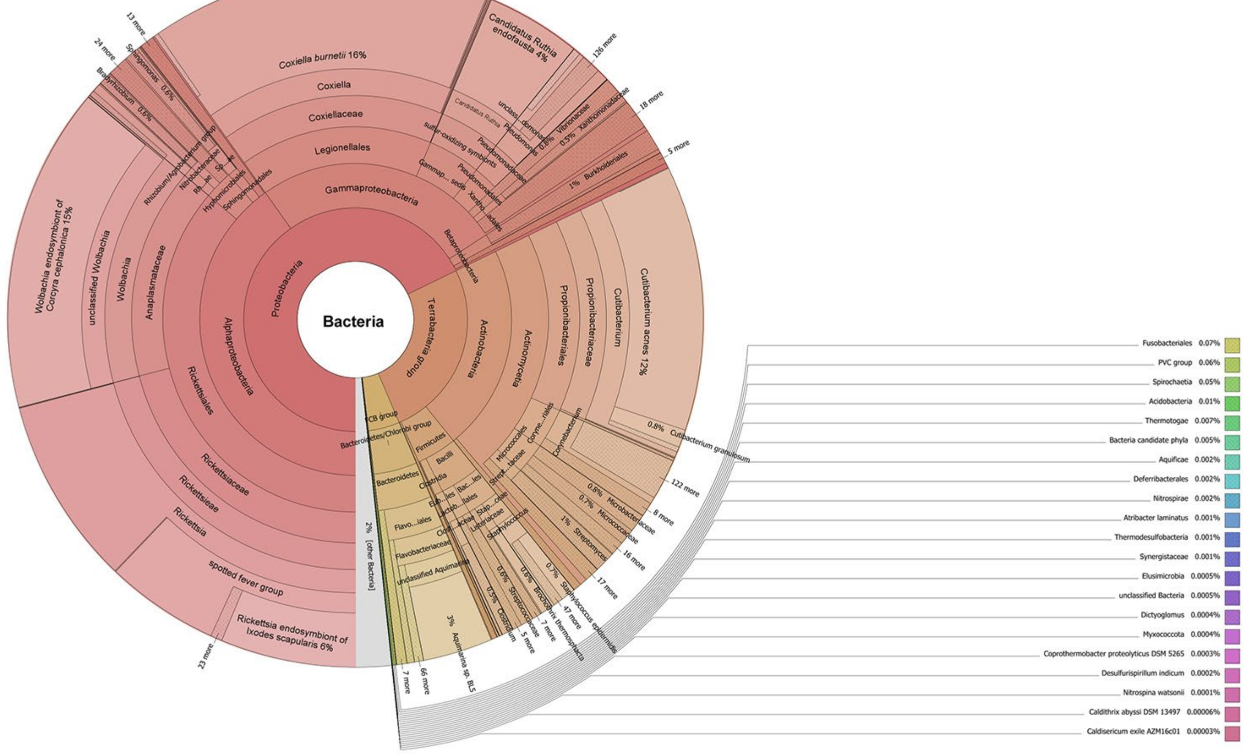

Appendix Figure 2. Analysis of the tick specimen's microbiome from the *Ixodes scapularis* tick parasitizing dog in Dawson County, Montana, USA, 2023. Chart summarizes a metagenomic scan of bacteria by using Kraken2 (*6*) and mapping to a database containing all viruses, bacteria, archaea, protozoa, and fungi available in RefSeq (https://www.ncbi.nlm.nih.gov/refseq) as of September 2024. Percentages correspond to the percentage of reads derived from bacteria that were uniquely assigned to that taxonomic level. Six percent of all bacterial reads (340,000 reads) mapped to the *Rickettsia* endosymbiont of *I. scapularis*. This bacteria sp. has been renamed *Rickettsia buchneri* (*7*) and is believed to be specific to *I. scapularis* ticks.
